# Coexistence of benign phyllodes tumor and invasive ductal carcinoma in distinct breasts: case report

**DOI:** 10.1186/2047-783X-17-8

**Published:** 2012-04-25

**Authors:** Guerino Barbalaco Neto, Claudia Rossetti, Natalia A Souza, Fernando LA Fonseca, Ligia Ajaime Azzalis, Virginia Berlanga Campos Junqueira, Vitor E Valenti, Luiz Carlos de Abreu

**Affiliations:** 1 Departamento de Mastologia, Faculdade de Medicina do 199 ABC, Av. Príncipe de Gales, 821, Santo André, SP, 09060-650, Brazil; 2 Departamento de Ciências Biológicas, UNIFESP, Rua Prof. Artur Riedel, Diadema, SP, 09972-270, Brazil; 3 Departamento de Morfologia e Fisiologia, Faculdade de Medicina do ABC, Av. Príncipe de Gales, 821, Santo André, SP, 09060-650, Brazil; 4 Departamento de Fonoaudiologia, Faculdade de Filosofia e Ciências, Universidade Estadual Paulista UNESP, Av. Higyno Muzzi Filho, 737, Marília, SP, 17525-900, Brazil

**Keywords:** Breast, Phyllodes tumor, Carcinoma

## Abstract

This report describes a rare case of coexistence of benign phyllodes tumor, which measured 9 cm in the right breast, and invasive ductal carcinoma of 6 cm in the left breast, synchronous and independent, in a 66-year-old patient. The patient underwent a bilateral mastectomy due to the size of both lesions. Such situations are rare and usually refer to the occurrence of ductal or lobular carcinoma *in situ* when associated with malignant phyllodes tumors, and more often in ipsilateral breast or intra-lesional.

## Background

Phyllodes tumors represent a rare biphasic neoplasm composed of epithelial and stromal elements, which corresponds to a total of 1% of breast cancers and around 2% to 3% of fibroepithelial tumors [1,2]. The concomitance of these tumors with epithelial malignant neoplasms is rare. The literature reported the association of phyllodes tumors with malignant epithelial components mainly in the form of ductal or lobular *in situ* lesions and less often in the invasive form. However, they are usually situated inside the lesion or near the fibroepithelial neoplasm tissue [3].

Treatment of these tumors depends on the epithelial and fibroepithelial lesion intrinsic profile. Excision of the lesion with margins is considered to be adequate in benign phyllodes tumors cases. On the other hand, when the phyllodes tumors are large, a simple mastectomy is recommended. In the case of epithelial injury, size, location and lymph node involvement are not considered in determining therapeutic approaches [3-5].

Carcinoma is the medical term for the most common type of cancer occurring in humans [6-8]. The synchronous coexistence of benign phyllodes tumor in one breast and invasive carcinoma in the other breast is rare. In fact, we found no reports in the literature documenting the association of phyllodes tumor and invasive ductal carcinoma. To our knowledge, this is the first report to describe a case of synchronous coexistence of benign phyllodes tumor and invasive ductal carcinoma in distinct breasts.

## Case presentation

**Figure 1:**
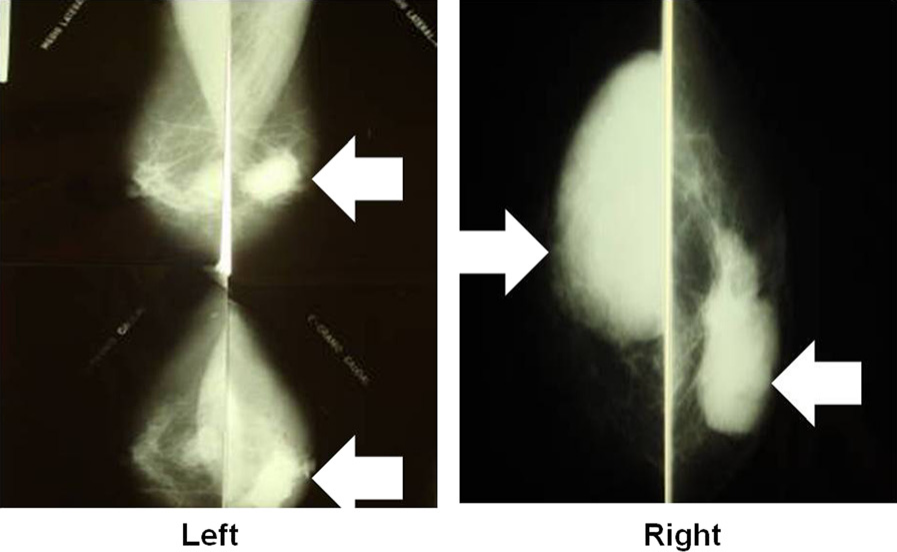
**“Mammographic examination of the patient.** Left 2006, 1 year later.

This is a 66-year-old patient, with four pregnancies, three normal deliveries and one abortion. She smoked from the ages of 10 to 51 years and had no family history of breast carcinoma. She reported the presence of progressively growing nodules in both breasts in 2005. In 2006, she underwent a mammography (Figure 1) with suspicious findings and was referred to the hospital. She did not visit the hospital for fear of a positive diagnosis. In December 2006, she underwent another mammography, and was examined again in February 2007. Mammography showed a regular 9-cm nodule in the right breast, without skin retraction, with elastic firm consistency, diffuse bulging breast and negative axillary lymph nodes. Mammography also noted a 6-cm nodule in the left breast with bulging and skin retraction located in inter-medial quadrants and retro areolar region, hardened, partially mobile and negative axillary lymph nodes. She was referred to preoperative assessment and surgery. Incisional biopsies of both breasts were performed. Biopsy of the left breast revealed the presence of invasive ductal carcinoma (pT3pn0, pMx), Nottingham grade II, nuclear grade III. Biopsy of the right breast showed a probable benign phyllodes tumor. No core needle biopsy was performed because the technology was not available in our clinic.

Based on the diagnosis and the size and location of the lesions, we decided to perform a simple mastectomy in the right breast and modified Madden mastectomy in the left breast.

**Figure 2:**
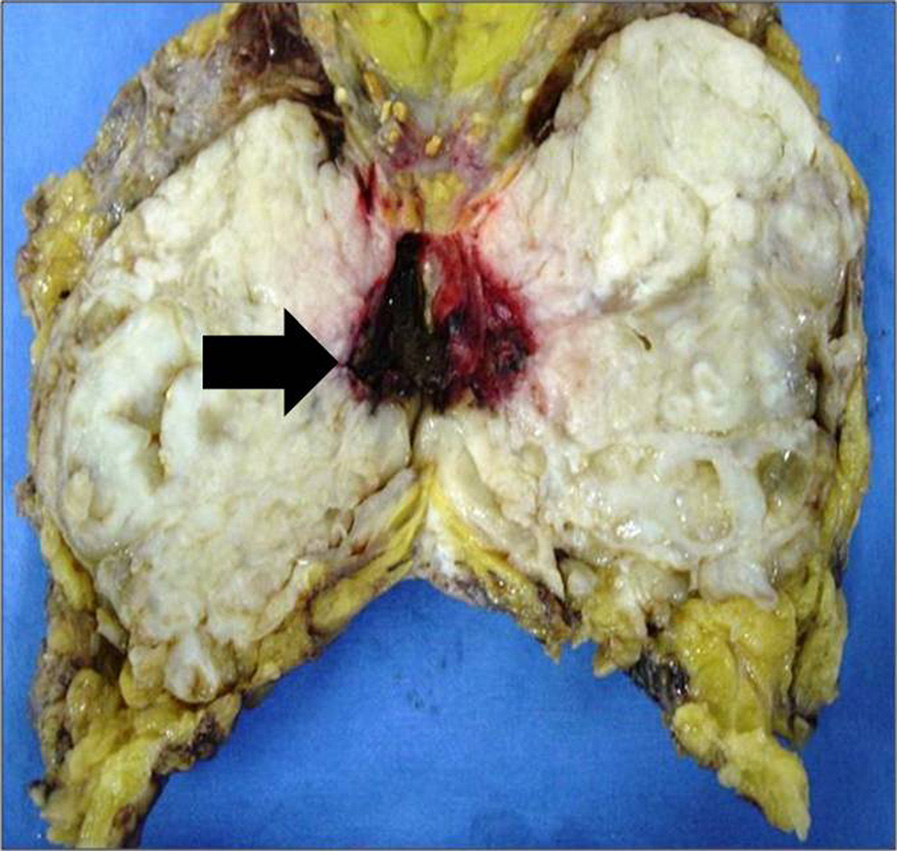
Anatomopathological results of phyllodes tumor.

**Figure 3:**
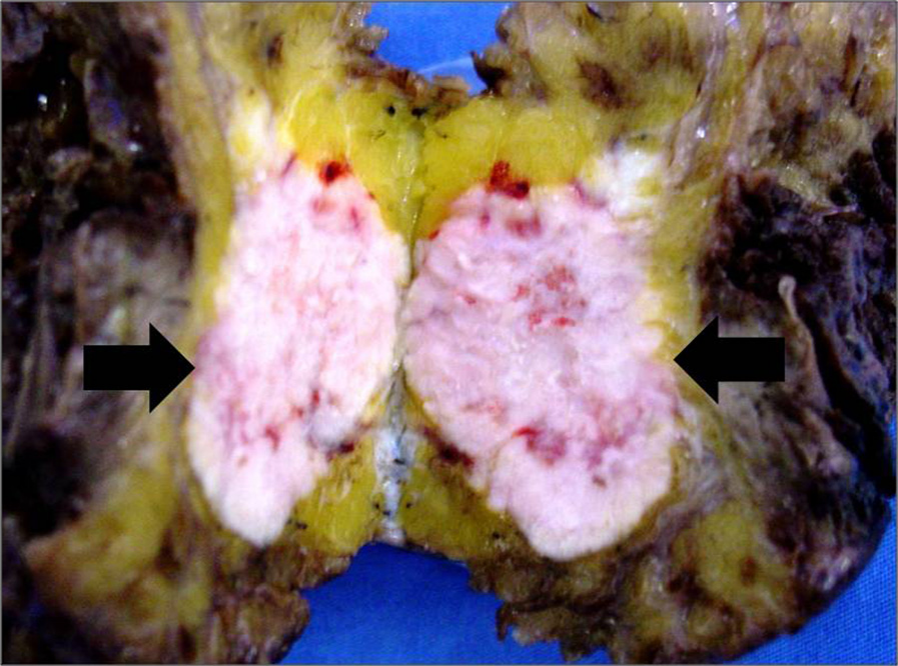
Anatomopathological results of the carcinoma in the left breast.

The anatomopathological final result showed the presence of benign phyllodes tumor in the right breast (Figure 2), which measured 9 cm in its longest axis, and invasive ductal carcinoma Nottingham grade II, nuclear grade III, which measured 7 × 6 cm in the left breast. Further evaluation indicated negative axillary lymph nodes and absence of metastasis (Figure 3).

For histological assessment, fragments were dipped in Gender liquid at 4°C for 24 h and were cut into small pieces of 1 mm^3^ and post-fixed in a 1% OsO4 solution for 2 h, dehydrated and embedded in araldite. Silver or gray thin sections (60 to 90 nm) were selected on a Porter-Blum MT-B ultramicrotome. The ultra-slices were mounted on copper silver grids with 200 patches and stained with uranyl acetate and lead citrate. The fragments were fixed in formaldehyde 10% at 4°C. Approximately 24 h later the fragments were cut in cryostat. This protocol followed the routine procedures of our laboratory [9-11].

**Figure 4:**
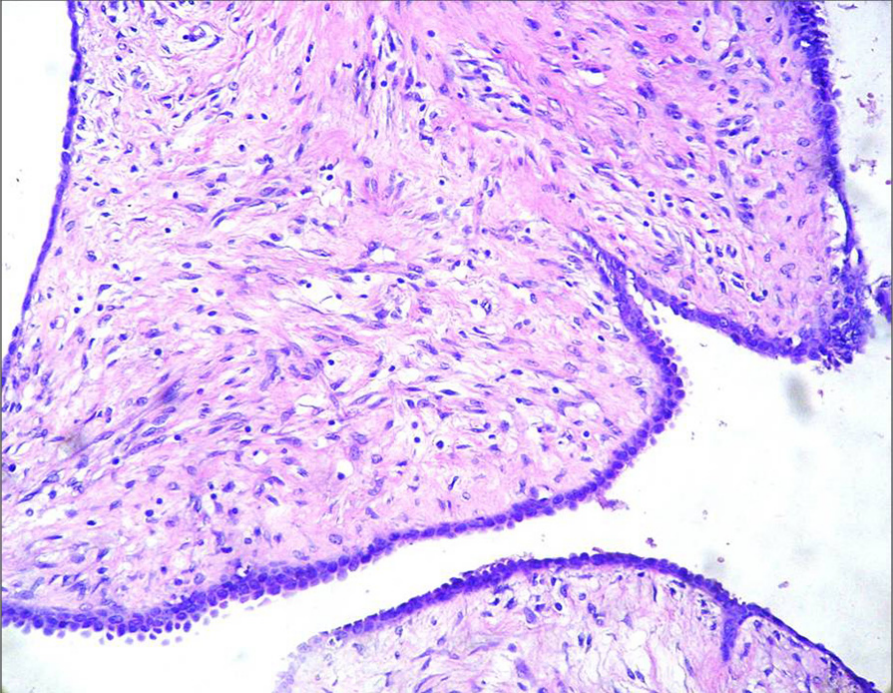
Histological results of phyllodes tumor.

**Figure 5:**
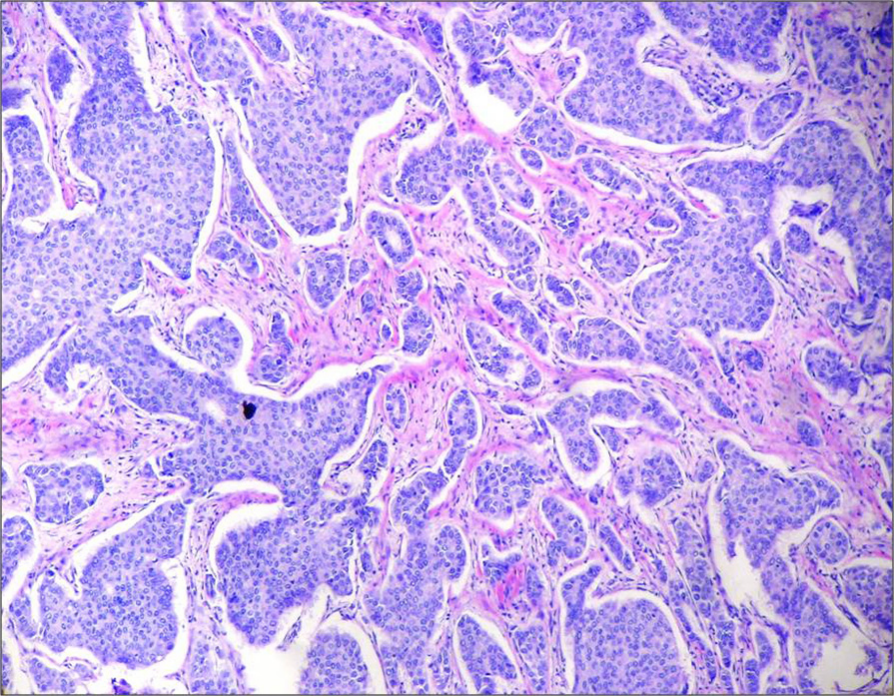
Histological results of the carcinoma in the left breast.

Figure 4 shows the histological evaluation of the of phyllodes tumor. Figure 5 shows the histological results of the carcinoma in the left breast focusing on steroid Her-2/neu-receptor. Preoperatively, history and physical exam were normal; bilateral mammography, abdominal and pelvic computed tomography (CT), usg total abdominal and pelvic, chest imaging, and biochemical tests (complete blood count, platelets, liver function tests, and alkaline phosphatase) were all normal. However, bone scan indicated a low probability metastases.

The patient was given adjuvant therapy composed of radiotherapy and chemotherapy for phyllodes tumor and carcinoma, respectively. Chemotherapy included six cycles of the FAC regimen (5-fluorouacil, doxorubicin, dyclophosphamide). To date, the patient has not shown any clinical or laboratory alterations.

## Discussion

Phyllodes tumor of the breast is classified in the literature as benign, borderline, or malignant subtypes based on features of the tumor such as necrosis, margins (pushing or infiltrative), cellular atypia, stromal overgrowth, and the number of mitoses per high power field [12,13]. Phyllodes tumors usually occur in women over 45 years of age, but may be present at any age, including in children [1]. Characteristics of the tumor which should be considered in evaluating its behavior and seriousness and to determine the proper treatment approach include: stroma profile, stromal cell atypia, growth pattern (heterologous or homologous), lesion borders (well demarcated or infiltrative), and presence or absence of necrosis [14]. For example, Mitotic activity lower than 5 per 10 high power fields suggests a benign behavior. Mitotic activity rates above 10 per 10 fields indicate potentially malignant behavior [15]. However, one criterion should never be evaluated independent of other criteria. Moreover, while a vast majority of these lesions present with benign features, approximately 30% exhibit malignancy stromal criteria [16]. Metastasis is usually hematogenous, described in around 13% in 10 years for malignant phyllodes tumors. Thus, axillary dissection is generally not recommended. Local recurrence is common even in benign forms, and was observed in more than 8% of cases in 10 years. Local recurrence is more common in borderline and malignant forms [17].

To our knowledge, this is the first report of a case of coexisting benign phyllodes tumor and invasive ductal carcinoma in distinct breasts. The association of carcinoma and phyllodes *in situ* tumor is rare. It has been reported that 81% of cases represent lobular *in situ* carcinoma, usually with intra-lesional location or on its adjacency. Around 33 such cases have been described in the literature. Other subtypes of cancer associated with phyllodes are related to tubular and squamous carcinoma [18]. The prognosis of carcinoma cases originating from phyllodes is generally favorable [18].

Synchronous and independent lesions in separate breasts have not been reported, especially benign phyllodes tumor, not associated with adjacent invasive lesions or intra-lesions. Some authors recommend treating phyllodes tumors based on their carcinomatous features [18]. In our case, we performed incisional biopsies of both breasts. As an adjuvant therapy, we administrated chemotherapy and radiotherapy for carcinoma and phyllodes tumor, respectively. With respect to phyllodes tumor, usually, surgical treatment alone is adequate. Some units proposed adjuvant chemo and radiotherapy in certain cases (recurrent phyllodes tumor after mastectomy, stromal overgrowth), but the role of adjuvant treatments is not yet clear. The treatment of phyllodes tumors depends fundamentally on surgery in the breast. A wide excision of the tumor with a 10-mm clear margin is recommended [19]; mastectomy is recommended if breast conservation surgery is not possible. Shelling out of tumors is not adequate, but frequently done because of the similitude with fibroadenomas and the difficulties of preoperative histological diagnosis [20].

Conservative surgery with excision margins (about 1 cm) showed a good prognosis for benign phyllodes with recurrence rates that range from 8% to 20% with no risk of metastasis. However, treatment noncompliance by patients, as reported in this case, is an important concern In this case, the choice of mastectomy became inevitable due to the large scale of the two lesions, a fact which unfortunately, also contributed to a poor prognosis.

Little is known about the genetic abnormalities in phyllodes. We could speculate that there is a genetic relationship between the two both lesions. Although oncogene activation and tumor suppressor gene inactivation are important mechanisms in the genesis, propagation, and spread of most cancers, the role of these processes in phyllodes tumor has not been previously explored. It is well known that allelic loss is a common early genetic alteration during tumorogenesis [21].

## Conclusions

The association of phyllodes tumor with breast carcinomas is rare, especially in the form of synchronous and independent lesions in separate breasts. We reported the first case of the coexistence of benign phyllodes tumor and synchronous, independent and invasive ductal carcinoma in separate breasts.

## Consent

Written informed consent was obtained from the patient for publication of this report and any accompanying images.

## Competing interest

The authors declare that they have no competing interest.

## Authors’ contributions

All authors approved the addition of the two new authors: LAA and VBCJ. GBN, CR, NAS, FLAF, LAA, VBCJ, VEV and LCdA participated in the acquisition of data and revision of the manuscript. All authors conceived of the study, determined the design, performed the statistical analysis, interpreted the data and drafted the manuscript. All authors read and gave final approval for the version submitted for publication.
